# DNA-encoded libraries (DELs): a review of on-DNA chemistries and their output

**DOI:** 10.1039/d0ra09889b

**Published:** 2021-01-19

**Authors:** Ying Shi, Yan-ran Wu, Jian-qiang Yu, Wan-nian Zhang, Chun-lin Zhuang

**Affiliations:** School of Pharmacy, Key Laboratory of Hui Ethnic Medicine Modernization, Ministry of Education, Ningxia Medical University 1160 Shengli Street Yinchuan 750004 China nxshiying@163.com; School of Pharmacy, Second Military Medical University 325 Guohe Road Shanghai 200433 China zclnathan@163.com

## Abstract

A DNA-encoded library is a collection of small molecules covalently linked to DNA that has unique information about the identity and the structure of each library member. A DNA-encoded chemical library (DEL) is broadly adopted by major pharmaceutical companies and used in numerous drug discovery programs. The application of the DEL technology is advantageous at the initial period of drug discovery because of reduced cost, time, and storage space for the identification of target compounds. The key points for the construction of DELs comprise the development and the selection of the encoding methods, transfer of routine chemical reaction from off-DNA to on-DNA, and exploration of new chemical reactions on DNA. The limitations in the chemical space and the diversity of DEL were reduced gradually by using novel DNA-compatible reactions based on the formation and the cleavage of various bonds. Here, we summarized a series of novel DNA-compatible chemistry reactions for DEL building blocks and analysed the druggability of screened hit molecules *via* DELs in the past five years.

## Abstract 2

We summarize a series of novel DNA-compatible chemistry reactions for DNA-encoded chemical library (DEL) building blocks and analyse the druggability of screened hit molecules *via* DELs in the past five years.

## Graphical abstract

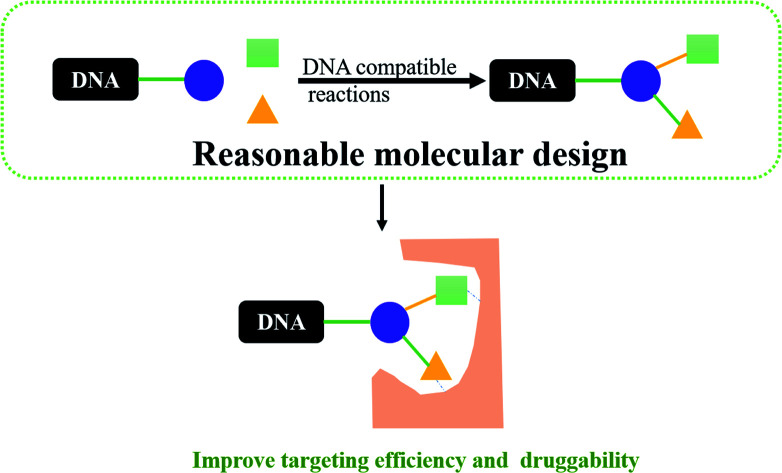

## 1 Introduction

**Fig. 1:**
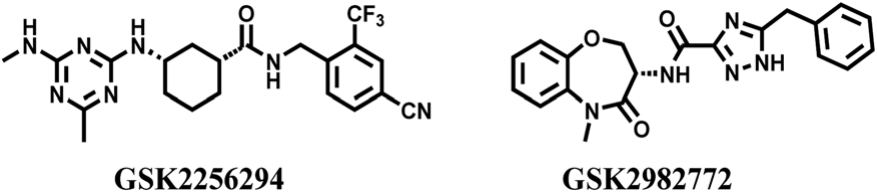
Compounds developed *via* DEL.

In 1992, Brenner and Lerner first proposed the concept of encoding a chemical library with sequenced nucleotide tags,^1^ and this concept was rapidly applied to practice by Janda and Brenner in 1993.^2^ In recent decades, DEL has become a technology platform that combines the advantages of chemical and biological display libraries. Every member in a DEL is constructed through polymerase chain reaction and DNA-compatible routine reaction. The identity of an individual compound can be determined *via* high-throughput DNA sequencing, because every molecule is correspondingly conjugated with its unique DNA barcode. DNA tags are used as barcodes, which ensure the high-precision hit screening and improve the application efficiency of chemical libraries. Library members can be completely stored in a minute space, and the trace amounts used for affinity capture procedures. Recently, DEL drown too much attention because of its unique advantages in drug discovery, which even catches up with traditional high-throughput screening (HTS), fragment-based drug discovery, phenotypic screening, *in silico* screening, and affinity selection through mass spectrometry.^3^ Compared with traditional approaches, DEL is more conducive to drug discovery and identification.^4^ Other advantages of DEL include (1) enormous library size, (2) small space for compounds storage, (3) a thimbleful of DNA-tagged molecules to affinity assay, (4) low-cost tools for academic institution and small pharmaceutical company, (5) and efficient collection of drug-like compounds.^5–7^ With the constant influx of capital, some clinical candidates are provided *via* DEL technologies in a short time.^3,8^ GSK reports the phase II clinical molecule, GSK2256294, developed through DEL, used to cure chronic obstructive pulmonary disease (Fig. 1).^9,10^ The GSK2982772, a receptor-interacting protein-1 kinase inhibitor developed by GSK, is applied to cure ulcerative colitis, rheumatoid arthritis, and psoriasis in phase II trials (Fig. 1).^11^

Recently, some excellent reviews have analysed the characteristics of DEL technology from different perspectives.^3,8,12,13^ Here, we have discussed the new exploration of the DEL-compatible chemistry and analysed the drug ability of the hit compounds isolated from DEL in recent selection campaigns. Finally, we have proposed our views on the current challenges and future directions for DEL.

## 2 Exploration of novel DNA-compatible reactions

The synthesis of functional molecules tagged with DNA played a considerable role in a promotion to the diversity of chemical spaces at the initial stage of the construction of DEL. Numerous catalysts should be developed and employed to promote the compatibility of the chemistry reactions that DNA participated. Solvents, temperature, and reaction time were screened and optimized. Here, we summarized the DNA-compatible reactions on the basis of the formation and the cleavage of various bonds, such as C–C, C–N, and C–O, in the recent five years.

### C–C sp^2^–sp^2^ coupling

Li successfully developed the DNA-compatible Suzuki–Miyaura reaction in aqueous media by using a water-soluble palladium precatalyst which could promote the coupling efficiency between DNA-linked aryl halides and boronic acids/esters (Table 1 entry 1).^14^ Results showed that phenyl chlorides were coupled with 70% boronates and the conversion rate was as high as 50%. Heteroaryl chlorides were more reactive than phenyl chlorides, and the conversion was over 50%. Aryl bromine and aryl iodide presented optimum reactivity, and the conversion achieved over 90%. Generally, the carbonylative reaction was accomplished under high concentration of carbon monoxide, which guaranteed that the carbon monoxide could insert into the palladium–electrophile complex efficiently.^15–17^ After the regulation of the reaction conditions based on their previous work, Li and his coworkers developed palladium-catalyzed DNA-linked aryl halides and generated aryl acid (Table 1 entry 2).^18^ The substrate was aryl and heteroaryl halides, and the typical carbon monoxide gas was changed to carbon monoxide sources, including *N*-formyl saccharin^19^ and molybdenum hexacarbonyl,^20^ which could decompose and generate carbon monoxide in the reaction system. After screening, molybdenum hexacarbonyl was chosen as the CO source. The result was consistent with those of previous works.^14^ (Hetero)aryl iodide and bromide substrates transformed into hydroxycarbonylation more efficiently than (hetero)aryl chloride. The hydroxycarbonylation reaction was successfully used to construct DEL, and the known hits for soluble epoxide hydrolase (sEH, EPHX2), a cardiovascular target, and L3MBTL1, a member of the malignant brain tumor family, were further validated. Liu's group first reported the Heck reaction on DNA.^21^ However, only three entries were described, and the conversion was moderate. The diversity of the substrates cannot be reflected. Recently, Dai and Lu described palladium-promoted DNA-compatible Heck reaction and optimized the reaction conditions, which were compatible for DNA-conjugated styrene/acrylamides and aryl halide^22^ (Table 1 entry 3). The substrates included aryl iodide, aryl bromide, and aryl borate/boric acid, and most of the observed conversion achieved over 60–95%. The possibility and the feasibility of constructing DEL were revealed. The Heck reaction was utilized to a three-cycle synthesis for DEL. The GlaxoSmithKline successfully and efficiently applied Pd(PPh_3_)_4_ to catalyze the Suzuki–Miyaura coupling reaction between DNA-conjugated phenyl bromides or phenyl iodides and pyridinyl bromides with (hetero)aryl boronic acids/esters.^23^ The aryl–aryl binding was generated, and the vast majority of the conversion achieved over 70% (Table 1 entry 4). Particularly, the furanyl, pyrazoleyl, and thiopheneyl boronic ester substrates underwent the coupling reaction, and the conversion reached 100%. Aryl iodide substrates coupled with DNA-conjugated halides more readily than aryl bromide substrates. Nevertheless, the substituted groups on the *ortho* position of aryl boronic acids/esters limited the formation of aryl–aryl bonds and reduced the conversion. Except (hetero)aryl iodide and bromide, (hetero)aryl chloride substrates were coupled with (hetero)aryl boronic acids/esters, and results demonstrated that Pd(PPh_3_)_4_ was unfit for the (hetero)aryl chloride-participated Suzuki–Miyaura coupling reaction with (hetero)aryl boronic acids/esters.^24^ After optimization, DNA-linked phenyl chlorides and pyridinyl chlorides were coupled with aromatic boronic acids/esters,^24^ which first used the newly developed palladium catalyst system (POPd) with ligand 1 in the Suzuki–Miyaura coupling reaction (Table 1 entry 5). Particularly, the pyrimidinyl chloride coupled with various boronates greatly expanded the chemical space in the DNA-encoded library. Lu also first developed the C–H activation reaction on DNA,^25^ and this reaction was catalyzed by ruthenium and applied in the reaction between DNA-linked acrylamides and aromatic acids (Table 1 entry 6). Recently, Lerner's group developed DNA-bound aryl fluorosulfonates to construct the C(sp^2^)–C(sp^2^) bond (Table 1 entries 7).^26^ Pd(OAc)_2_ as the catalyst was used in Suzuki–Miyaura cross-coupling reactions where aryl boric acid and aryl alkyne participated in. Waring's team developed a new and efficient method for the construction of aryl C–C bonds through the Suzuki–Miyaura reaction. The DNA-tagged phenyl iodine, aryl or heteroaryl boric acid/boronate esters, and pinacolato were locked in commercial micellar surfactants (Table 1 entry 8). This new methodology avoided the detectable remarkable DNA degradation, and even improved the conversion which the most yield of the coupling reactions were near to 100%. After the condition optimization, the methodology in the synthesis of DEL was used.^27^

**Table 1:** C–C sp^2^–sp^2^ coupling DNA-compatible reactions

Entry	DNA-compatible reactions	Conditions
1	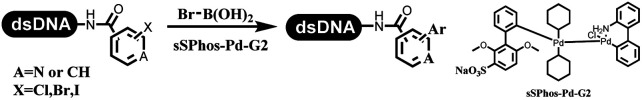	CsOH/H_2_O, 80 °C, 15 min
2	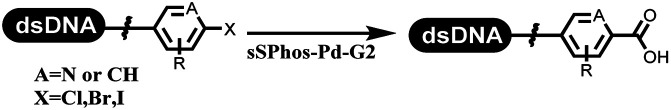	CsOH, Mo(CO)_6_, 80 °C, 15 min
3	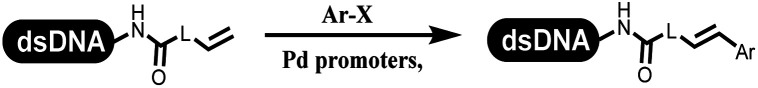	Base, organic solvent/H_2_O, 80 °C/6 h
4	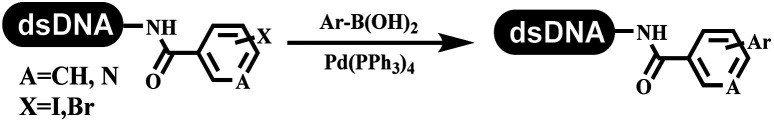	Na_2_CO_3_, H_2_O, 80 °C
5	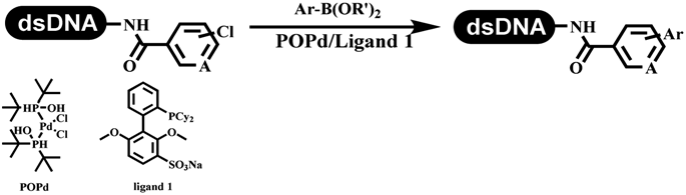	KOH/H_2_O
6	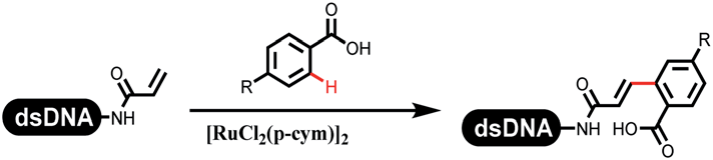	KOAc buffer/DMF, 60 °C, 10 h
7	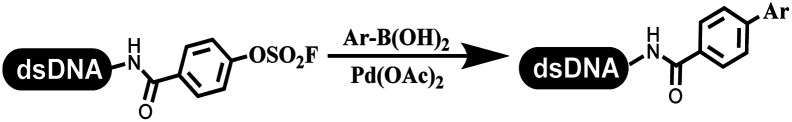	Suzuki–Miyaura
8	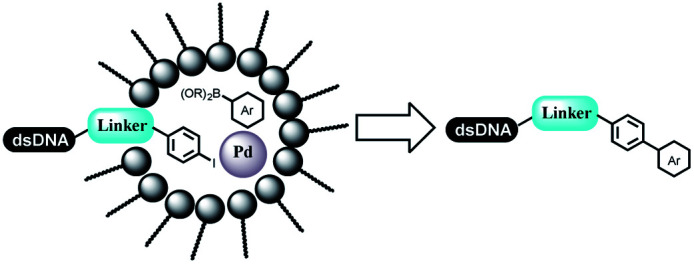	K_3_PO_4_, (800 equiv.), 2% TPGPS–750 M, 15% THF, 60 °C

### C–C sp^2^–sp and C–C sp^2^–sp^3^ coupling

Lerner's group developed DNA-bound aryl fluorosulfonates which was used to construct the C(sp^2^)–C(sp) bonds (Table 2 entry 1).^26^ Neri's group achieved the Sonogashira cross-coupling reaction on DNA, in which the DNA-linked phenyl iodine was coupled with (het)aryl, guanidyl, and aliphatic alkynes under the (PdCl[allyl])_2_ catalysis (Table 2 entry 2).^28^ Nearly half of the reactions worked, and the conversion achieved 75%. Yu's team exploited the C(sp^3^)–H activation on DNA.^29^ The DNA-linked aryl iodides reacted with the β-position C(sp^3^) of aliphatic carboxylic acids, amides, and ketones in water, and this reaction was catalyzed by palladium catalysis (Table 2 etrentry 3). The structurally diverse substrates were compatible with DNA, which contained enriched C(sp^3^) character, chiral centers, cyclopropane, cyclobutane, and heterocycles. Recently, Peng's team developed Suzuki–Miyaura cross-coupling on DNA,^30^ in which DNA-linked aryl bromides reacted with potassium Boc-protected amino methyl trifluoroborate and finally formed benzylamine under the Pd(OAc)_2_ catalysis (Table 2 Entry 4). Peng's team optimized the cross-coupling condition and preferred the combination of ligand and base (rac-BIDIME and K_2_CO_3_). The DNA-conjugated substrates comprised diverse (het)aromatic bromides, and most conversion achieved 70%. Pfizer and HitGen Inc. Implemented the photoredox with nickel and iridium catalysis between decarboxylated α-amino acids and DNA-lined aryl halides (iodide and bromine) in aqueous solution with blue LED (Table 2 Entry 5).^31^ The methodology possessed huge potential for the preparation of DEL because of the mild reaction conditions on DNA. GSK developed Ni/photoredox-catalyzed C(sp^2^)–C(sp^3^) cross-coupling and used the photoredox catalysis in radical/polar crossover alkylation for the construction of DEL.^32^ Ni/Photoredox promoted DNA-linked (hetero)aryl halides coupled with alkyl–DHPs and α-amino acids in 49 examples, and the overall conversion was 40–80%. The photoredox catalyst catalyzed DNA-linked aryl trifluoromethyl alkene radical/polar crossover alkylated with alkyl silicates, DHPs, and α-amino acids, and almost all conversions were over 60% (Table 2 Entry 6). Molander's group found new radical precursors generated from primary or secondary alkyl bromides and α-silylamines, and used the radical precursors to couple with DNA-conjugated (het)aryl bromides and iodides to form the target molecules under the Ni/photoredox dual catalyst (Table 2 entries 7–9).^33^ Baran's team used nickel to mediate the decarboxylative C(sp^2^)–C(sp^3^) cross-coupling under the conditions of reversible adsorption to solid support, which was compatible with DNA (Table 2 Entry 10).^34^ After optimization, the conversion was above 80%. The condition was suitable for substrates containing phenyl, saturated cycloalkyl, and N-heterocycle, which reacted with the DNA-bound phenyl iodine. Liu's team first reported a new and highly efficient method for constructing C3-alkylated indole structures on DNA.^35^ At the beginning, the DNA-linked indole reaction with aldehydes forming the products in two steps under the metal-free catalysis was explored. Most conversions achieved 70–94% (Table 2 entry 11). The aldehydes were replaced with DNA-linked moieties, and results indicated that the mild conditions promoted the current reactions. The conversions improved heavily (Table 2 entry 12).

**Table 2:** C–C sp^2^–sp and C–C sp^2^–sp^3^ coupling DNA-compatible reactions

Entry	DNA-compatible reactions	Conditions
1	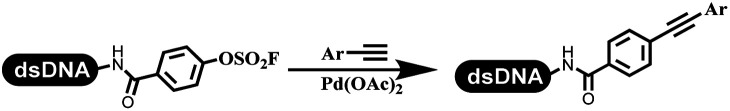	Sonogashira cross-coupling
2	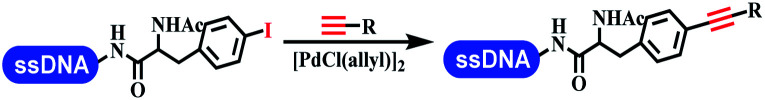	Na_2_CO_3_/H_2_O
3	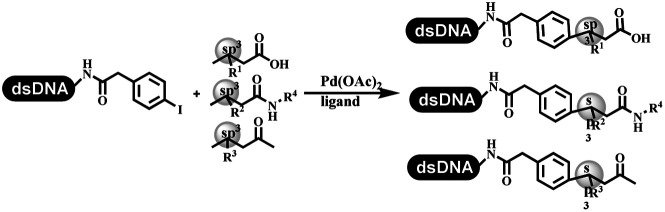	AgTFA
		NaOAc
		H_2_O/DMA
		80 °C, 20 h
4	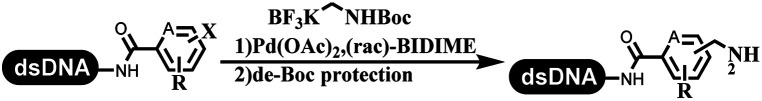	K_2_CO_3_, DMAc/H_2_O, 95 °C, 2 h
5	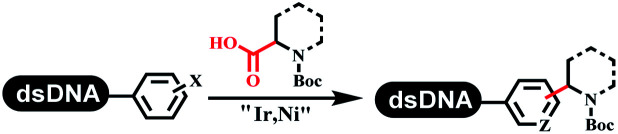	K_2_HPO_4_, DMSO/H_2_O, RT
		Blue LED
6	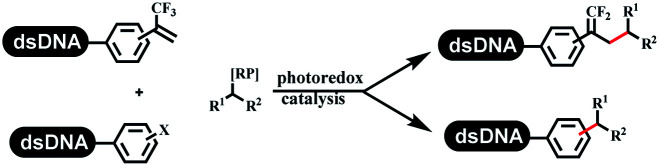	DMSO/H_2_O, RT
		Blue LED
7	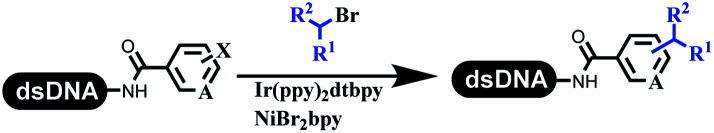	Et_3_N, DMSO/H_2_O
		Kessil lamp, 45 min
8	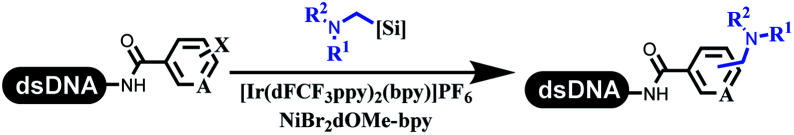	DMSO/H_2_O
		Kessil lamp, 15 min
9	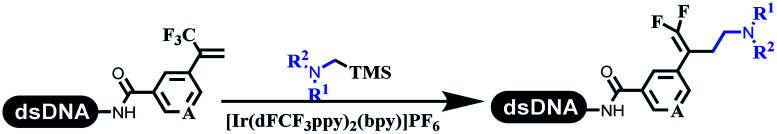	DMSO/H_2_O
		Kessil lamp, 5 min
10	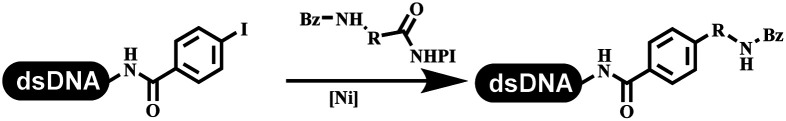	RASS, K_2_CO_3_, DMA
11	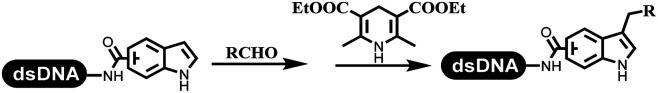	9DMSO/H_2_O, NaOH, 60 °C
12	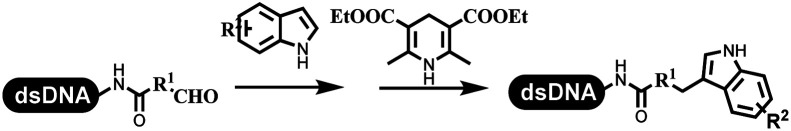	DMSO/H_2_O, RT

### Construction of the C–C sp^2^–sp^2^ and the C–C sp^3^–sp^3^ bonds

Dai's group synthesized pyridazines on DNA through inverse electron demand Diels–Alder (IEDDA) reactions (Table 3 entry 1). The DNA-linked tetrazine reacted with alkene which contained terminal olefin and cyclo-olefin in aqueous solutions, and the reaction was catalyzed by copper(ii). The DNA-compatible reactions included Suzuki–Miyaura coupling, acylation, and S_N_Ar substitution reactions, which the conversion was near to 100%.^36^ Xu and his coworkers used copper(ii) to catalyze the tetrazine-mediated IEDDA reactions on DNA (Table 3 entries 2 and 3). Tetrazine reacted with DNA-tagged terminal olefin or cyclo-olefin and resulted in 90% of the desired products.^37^ Grubbs Ru reagents were widely used in ring-closing metathesis (RCM) for drug discovery.^38–42^ Recently, Lu and his coworkers first used Grubbs Ru reagents to promote DNA-linked RCM and cross-metathesis reactions.^43^ After conditions optimization, the conversion of the closing metathesis reaction on DNA achieved 50–85% (Table 3 Entry 4). Mg^2+^ prevented the decomposition of the DNA. Simmons' team used the Grubbs third-generation catalyst B to construct a new olefin through RCM reactions,^44^ and the average conversion was 41% (Table 3 Entry 5). In 2018, Dai's group explored proline-catalyzed IEDDA among DNA-tagged tetrazine, ketones, and aldehydes.^36^ Results showed that the average yield was 69% (Table 3 Entry 6). Peng's team synthesized the DNA-linked α,β-unsaturated carbonyl compounds *via* the intermolecular Wittig olefination reaction.^45^ They explored the catalysis of phosphine reagents, and chose PPh_2_CH_3_. KH_2_PO_4_ and DMAc as the preferred additive and solvent, which promoted the conversion neaer to 72%. DNA-conjugated α-chloroacetamides reacted with (het)aromatic and aliphatic aldehydes, the results indicated that the conversion for (het)aromatic aldehydes preferred the aliphatic aldehydes (Table 3 entry 7). For DNA-conjugated aldehyde reacting with α-halo acetamides or ketones, most conversions achieved approximately 60–90% (Table 3 entries 8 and 9). Baran's team constructed the C(sp^3^)–C(sp^3^) bond through zinc nanopowder-mediated Giese addition reaction on DNA-linked molecules, which was based on the radical mechanism (Table 3 entry 10).^46^ Some highly hindered C(sp^3^)–C(sp^3^) linkages were also synthesized, and substrates containing amino, carboxyl, and dipeptide were compatible for the construction of DEL. In the past few years, the photocatalysis was utilized to synthesize drugs and key pharmaceutical intermediates.^47^ Merck implemented photocatalysis in small-scale batch reactions.^48^ Lilly used photoredox catalyst to synthesize the key intermediate of the JAK2 inhibitor LY2784544.^49^ Recently, Pfizer achieved the addition reaction between decarboxylated α-amino acids and DNA-linked Michael receptor under mild conditions by photoredox with iridium catalyst^50^ (Table 3 entry 11). They screened the reaction conditions found that photoredox catalyst and the light were necessary which determined whether the reaction happened or not. Proline catalyzed DNA-tagged aldehydes react with soluble ketones for asymmetric aldol reaction (Table 3 entry 12).^51^ The conversions of most reactions were up to 90% with 70% e.e. The formed β-hydroxy ketones were used as substrates for the Mitsunobu reactions, and results showed that the conversions of all reactions were 90–99% with 57–98% e.e. Two years later, Dai's group developed a convenient and efficient formal [4 + 2] cycloaddition reaction, which was compatible with DNA.^52^ This reaction was utilized to construct the diverse thiazole-fused dihydropyrans (Table 3 entry 13). The solvents were screened, and results showed that the combination of DMSO and water could improve the conversion perfectly. The DNA-tagged multisubstituted thiazol-4(5*H*)-one worked efficiently with aliphatic and (het)aryl aldehydes or cycloalkyl ketones under the pyrrolidine/BzOH catalyst.

**Table 3:** Formation of the C–C sp^2^–sp^2^ and the C–C sp^3^–sp^3^ bonds

Entry	DNA-compatible reactions	Conditions
1	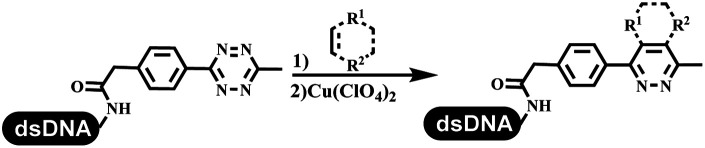	(1) DMSO, H_2_O
		(2) bypridine
		TEMPO
2	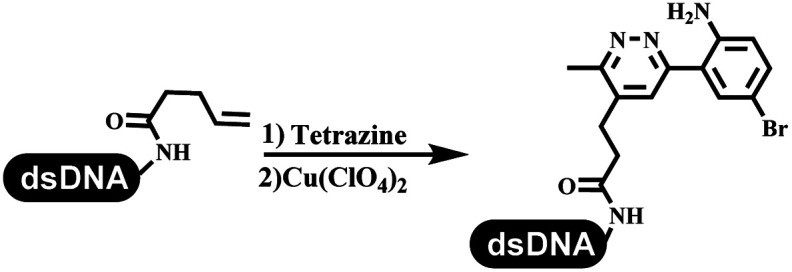	(1) DMSO, H_2_O
		(2) bypridine
		TEMPO
3	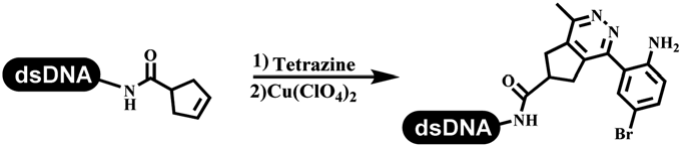	(1) DMSO, H_2_O
		(2) bypridine
		TEMPO
4	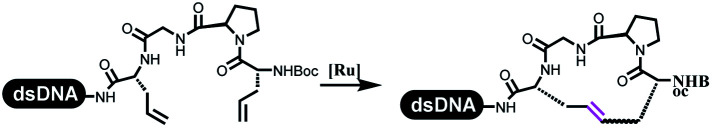	H_2_O : *t*-BuOH = 3 : 2
5	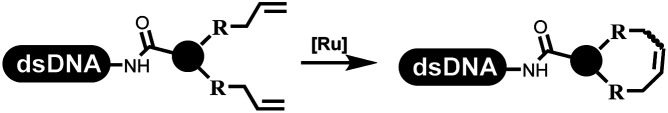	H_2_O : EtOH : MeOAc = 5 : 4 : 1
6	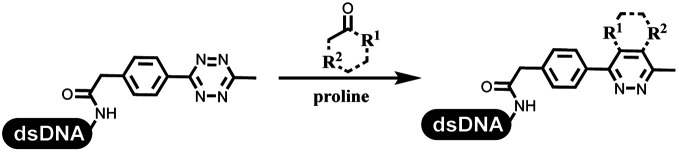	DMSO/water, 20 °C
7	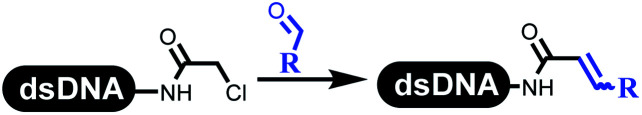	PPh_2_CH_3_, KH_2_PO_4_
		pH 9.45 buffer
		80 °C, 6 h
8	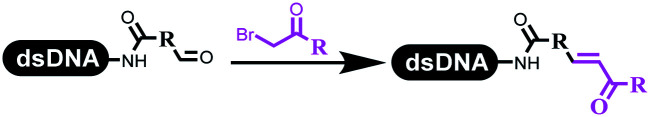	PPh_2_CH_3_, KH_2_PO_4_
		Sodium borate buffer/DMAc
		80 °C, 2 h; 25 °C, 4 h
9	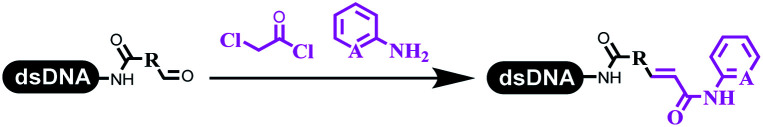	PPh_2_CH_3_, KH_2_PO_4_
		Sodium borate buffer/CH_3_CN
		80 °C, 2 h; 25 °C, 4 h
10	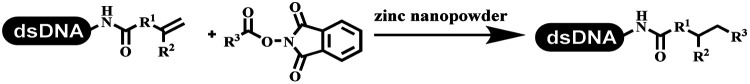	pH 5.5–6.5 buffer/DMSO, RT, 2 h
11	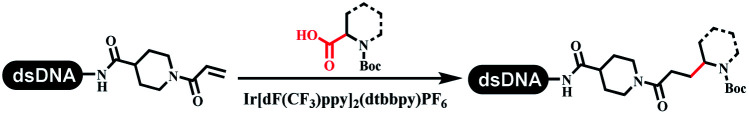	K_2_HPO_4_, DMSO/H_2_O, RT
		Blue LED
12	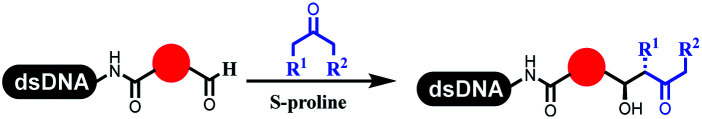	DMSO, RT, 16 h
13	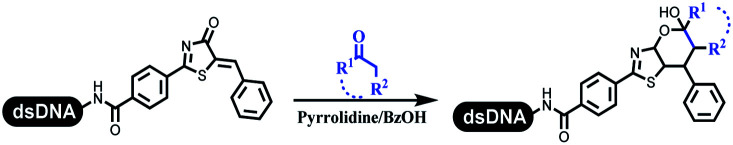	DMSO/H_2_O, RT

### Construction of C(sp^2^)–X (X = N, O, P, S, Se) bonds

The C–N coupling appeared later than the C–C coupling for DNA-conjugated aryl halides. Lu and his coworkers first successfully developed C–N coupling reactions on DNA-linked aryl iodide and aromatic amines, and these reactions were catalyzed *via* the Buchwald *t*-butyl-XPhos precatalyst G1 (Table 4 entry 1).^53,54^ Two years later, Torrado first reported the palladium-catalyzed C–N coupling between DNA-conjugated aryl bromides and aromatic amines, which were successfully utilized in the production of the third cycle of DEL (Table 4 entry 2).^55^ Recently, Lerner's group developed DNA-bound aryl fluorosulfonates to construct the C(sp^2^)–N bonds, which belongs to Buchwald–Hartwig cross-coupling reactions (Table 4 Entry 3).^26^ T-BuBrettphos Pd was utilized in the cross-coupling reaction, and another substrate was substituted with aryl amines. The conversions of all cross-coupling reactions were about 80–100%. Simmons' group recently developed new DNA-compatible conditions for the formation of the C–N bond.^56^ The DNA-tagged aryl halogen (*i.e.*, Cl, Br, and I) coupled with anilines and 2° amines under *N*-heterocyclic carbene–palladium catalyst, which was used to construct the DEL that contained 63 million molecules (Table 4 entry 4). Copper-catalyzed C–N coupling reactions had a long application history in drug discovery.^57,58^ Nevertheless, copper-catalyzed reactions for the construction of DEL appeared until 2017, which Lu first reported the copper-catalyzed Ullmann N-arylation of amino acids and aliphatic primary amines with aryl iodide on DNA.^53^ Cu(i) combined with amino acids, which also acted as the ligand, could promote C–N coupling reactions. Simultaneously, the copper interacted with DNA and enabled DNA decomposition without amino acid. The CuSO_4_: proline (1 : 2) complex efficiently catalyzed the C–N coupling between aliphatic primary amino and DNA-conjugated aryl iodide (Table 4 entry 5). Berst used Cu(OAc)_2_ and a new ligand L15 complex not only to catalyze the same reaction as Lu's, but also promoted various hindered second amines coupled with the DNA-conjugated aryl iodide (Table 4 entry 6).^59^ Except C–C coupling, the construction of C–N bond was mediated by nickel catalyst. The DNA linked phenyl iodine could react with alkylamines and heterocyclyamines, and the result showed that the conversion of alkylamines was more preferred (Table 4 entry 7). Recently, Dawson and his coworkers developed new methodologies to synthesize C–S and C–P bonds on DNA (Table 4 entries 8 and 9).^60^ The DNA-linked (het)aryl iodide reacted with aryl, heteroaryl, and alkyl thiols, which was catalyzed by nickel. At the same time, phosphinic chlorides were competent coupling partners for aryl iodides to construct C–P bond. Zhang's team developed the nontransition metal-mediated formation of C–O and C–S bonds.^61^ DNA-conjugated heteroaryl quaternary ammonium salt reacted with aliphatic and arylanol or the mercapto compounds to form the designed molecules under mild conditions. Most conversions achieved above 70% (Table 4 entries 10 and 11). Lerner's team constructed the C–Se bond off DNA, which was catalyzed by rhodium(iii). After optimization, they transferred the method to on-DNA reaction (Table 4 entry 12).^62^ The DNA-tagged indole derivatives reacted with benzoselenazolones, which were substituted with halogen (*i.e.*, F, Cl, and Br) and methoxy in phosphate buffer–DMA (7 : 1) under (RhCp^*^[MeCN]_3_[SbF_6_]_2_) catalysis. The yield of monosubstituted products was better than that of multisubstituted products.

**Table 4:** Formation of C(sp^2^)–X (X = N, O, P, S, and Se) bonds on DNA

Entry	DNA-compatible reactions	Conditions
1	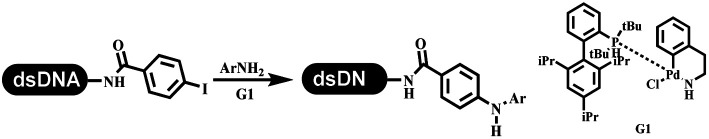	CsOH, DMA, 100 °C
2	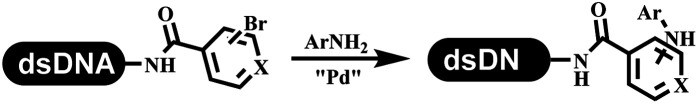	Water, DMA, NaOH
3	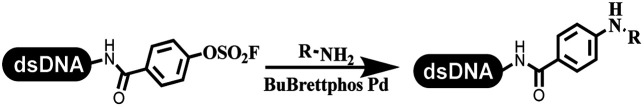	Buchwald–Hartwig cross-coupling
4	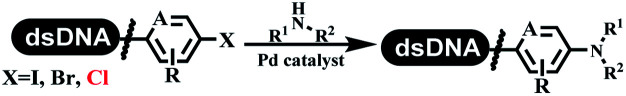	DMA, CsOH, 80 °C-95 °C
5	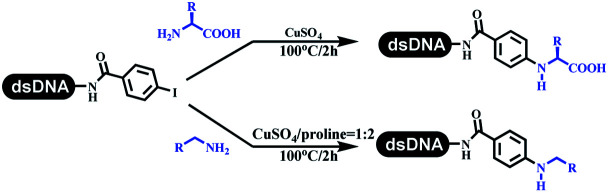	KOH, sodium ascorbate, H_2_O, DMA
6	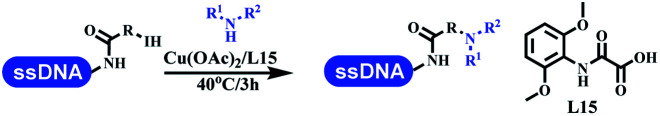	Sodium ascorbate, K_3_PO_4_, DMSO/water, 40 °C, 3 h
7	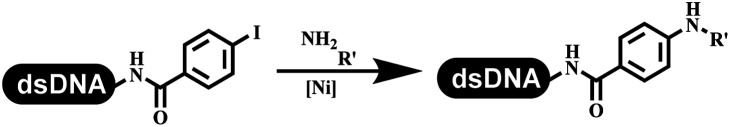	RASS, DMA, MS
8	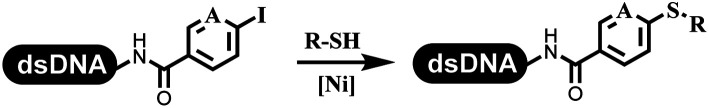	K_2_CO_3_, DMA
9	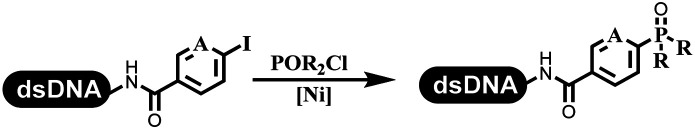	Phosphonic chloride, 4,4′-di-tertbutyl bipyridine
10	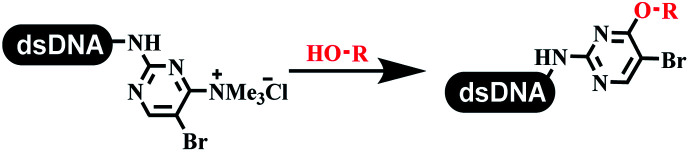	K_2_CO_3_ or KOH, DMA + H_2_O, RT or 60 °C
11	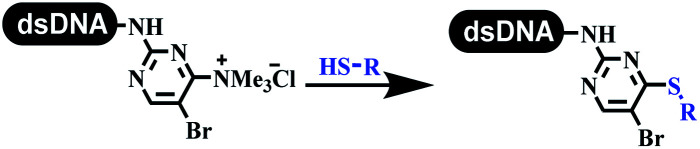	K_2_CO_3_, DMA + H_2_O, RT or 80 °C
12	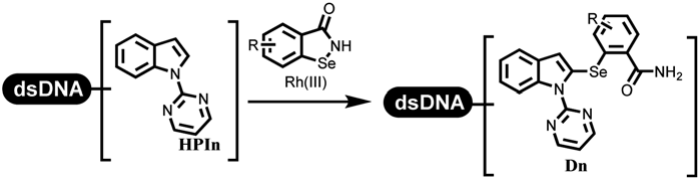	DCE, 100 °C, 1 h

### Multicomponent reaction

Brunschweiger's group developed multicomponent reactions on DNA which was catalyzed *via* various transition metal catalysts. They reported the Petasis 3-component reaction on DNA, which was catalyzed *via* copper(i)/bipyridine (Table 5 entry 1). The yield of most products were above 70%, and the *R*^2^ was confirmed as phenyl which benefited the reaction.^63^ Recently, they reported oligothymidine-initiated DNA-encoded chemistry,^64^ which described a hexathymidine oligonucleotide (hexT)-linked group reacted with other components. This process was catalyzed by Au(i), and the synthesis steps were recorded by coding DNA sequences. They optimized the BB, developed Au(i)-catalyzed 3-component reactions on DNA, and constructed the DNA-conjugated spiroheterocycles from either DNA-coupled aldehydes, hydrazides, or alkynols (Table 5 entry 2).^65^ Thymine-, cytosine-, and adenine-containing DNA were used in the reaction. Additionally, they synthesized the DNA-conjugated isoquinolones *via* the Yb(iii)-mediated Castagnoli–Cushman reaction under anhydrous conditions (Table 5 entry 3). The conditions of the Castagnoli–Cushman reaction were optimized, and the formation of isoquinolones was summarized. Aniline (500 equiv.) in dichloromethane/triethyl orthoformate (2 : 1), Yb(OTf)_3_ (50 equiv.), and homophthalic anhydride (500 equiv.) in dichloromethane were used.^66^ The 1,3-dipolar cycloaddition was conventionally used to synthesize 5-membered heterocyclic compounds. Their team developed silver-mediated (1,3)-cycloaddition to synthesize highly substituted DNA-conjugated pyrrolidines (Table 5 entry 4).^66^ The reaction conditions were optimized using 1000 equiv. aldehydes, 100 equiv. AgOAc, 1000 equiv. dipolarophiles, and 1000 equiv. triethylamine in ACN/triethyl orthoformate (2 : 1). The conversion achieved about 50%. They constructed the 6-membered nitrogen heterocycle through the ZnCl_2_-mediated aza-Diels–Alder reaction on DNA^67^ and screened the ratio among the DNA-tagged aromatic aldehydes, amines, Danishefsky's diene, and ZnCl_2_, which revealed that the most suitable ratio was 1 : 500 : 500 : 50. Among seven kinds of anhydrous organic solvents, acetonitrile, in which a conversion of 82% was achieved (Table 5 entry 5). Brunschweiger's group also developed isocyanide multicomponent reactions on DNA (Table 5 entries 6–9).^68^ DNA–aldehyde conjugates reacted with a diverse set of isocyanides, carboxylic acids and amines, that formed the products *via* the Ugi 4-component reaction (U-4CR) with nearly full conversion. DNA-linked aldehydes reacted with isocyanides, amines, and TMSN_3_ to form azide derivatives *via* the Ugi-azide 4-component reaction. Nearly all combinations of substrates transformed the designed molecules with high conversion. Another U-4CR/aza-Wittig reaction was utilized to synthesize the oxadiazole core under similar conditions as the U-4CR. Finally, the Groebke–Blackburn–Bienaymé 3-component reaction was completed on DNA. After the optimization of catalysts, the AcOH was preferred, and the average conversion achieved 63%.

**Table 5:** Multicomponent reaction on DNA

Entry	DNA-compatible reactions	Conditions
1	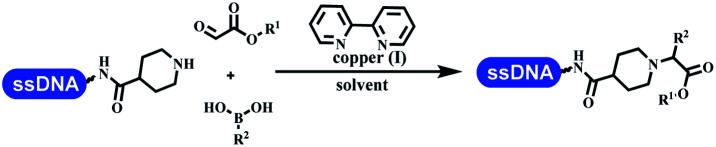	DMF/TEOF, 50 °C, 24 h
2	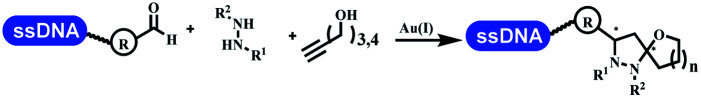	THF, RT, 20 h
3	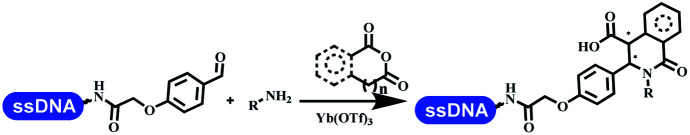	CH_2_Cl_2_/triethyl orthoformate, RT, 4 h
4	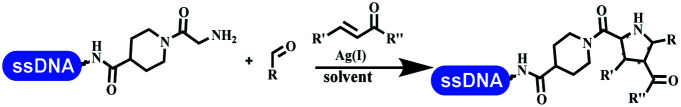	ACN/triethyl orthoformate, RT, 6 h
5	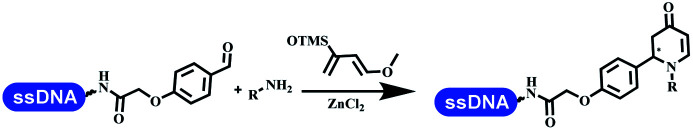	MeCN/TEOF, RT, 4 h; MeCN, RT, 1 h; NH_3_, 50 °C, 6 h
6	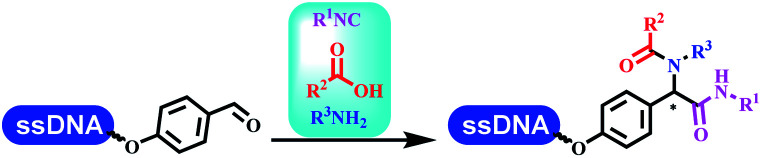	Ugi 4-component reaction
7	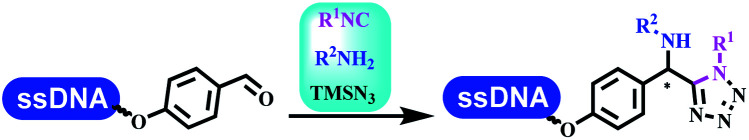	Ugi–Azide 4-component reaction
8	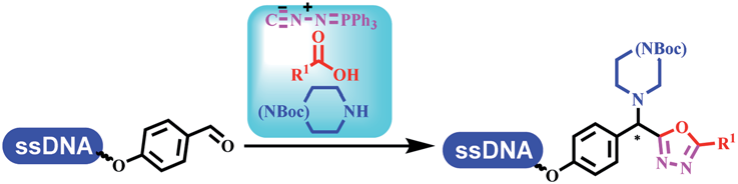	Ugi 4-component/aza-Wittig reaction
9	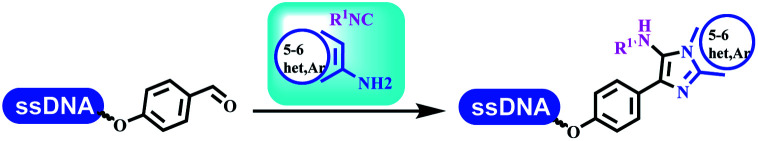	Groebke–Blackburn–Bienayme 3-component reaction
10	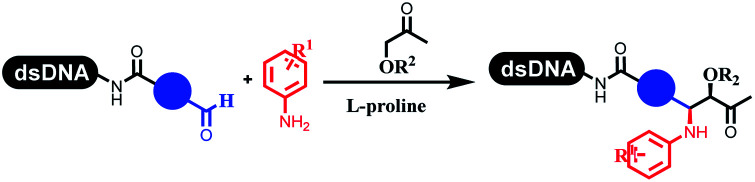	DMSO, RT, 18 h
11	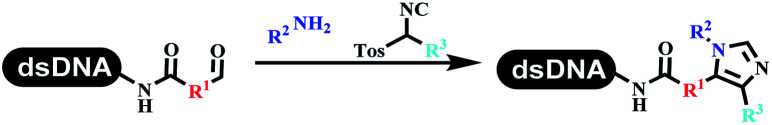	Morpholine,aq. DMA, 45 °C

Kodadek's group developed the asymmetric Mannich reaction between DNA-linked aldehydes, soluble ketones and anilines, which was catalyzed by proline.^69^ The conversion of most reactions was 65%. The substituted group on the *para* position of anilines could accelerate the formation of the products, and the average conversion was above 95% (Table 5 entry 10). Satz's team first reported the new synthesis strategy for the imidazole *via* the one-pot Van Leusen 3-component reaction on DNA (Table 5 entry 11).^70^ Organic bases improved the conversion more efficiently than inorganic bases. Additionally, the ratio of the organic solvent to water was a significant factor to the reaction, which indicated that 62% DMA was preferred. Finally, mild heating (45 °C) could afford high conversion. Aldehyde–DNA conjugates reacted with (het)aryl, aliphatic (cyclo)alkyl primary amines, and various commercial toluenesulphonylmethyl isocyanide molecules, and most conversions achieved 90%.

### Ring-closing and ring-opening reactions

Neri's group synthesized triazoles through DNA-tagged phenylalanine-based scaffold containing an azido group, (het)aryl, guanidyl and aliphatic alkynes, which was catalyzed *via* Cu(OAc)_2_ (Table 6 Entry 1).^28^ More than half of the reactions worked, and the conversion was up to75%. Peng's team synthesized 1,2,3-triazoles *via* an efficient DNA-compatible reaction. DNA-conjugated alkynes, aryl borates, and TMS-N3 conducted a click cycloaddition reaction in a one-pot reaction that was mediated by copper(ii) (Table 6 entry 2).^71^ Schreiber's group developed [2 + 2], [3 + 2], and [4 + 2] reactions on DNA.^72^ The DNA-tagged silyl derivatives underwent the cycloaddition reaction with 1,3-dipoles, olefins, and *N*-substituted pyrroles under the CsF catalysis. The yield of most products was above 90% (Table 6 entry 3). Dominik's group constructed new C(sp^3^)–C(sp^3^) bonds, and DNA-tagged cyclobutanes were formed on a photocatalytic [2 + 2] cycloaddition reaction in aqueous solution. This reaction was catalyzed by the iridium-based photocatalyst, Ir(ppy)_2_(dtbbpy)PF_6_ (Table 6 entry 4). Ketones were more readily formed than esters, and the heterocyclic substituted cinnamates were consumed slower than the phenyl cinnamates, which resulted in different d.r values.^73^ They used the method to construct a three-cycle DNA-encoded library. A year later, Liu's team and their coworkers successfully synthesized the multifunctional 2-aminobenzimidazoles on DNA *via* the iodine-promoted cyclization.^74^ 2-Aminobenzimidazoles were synthesized through the thiourea formation and the I_2_-promoted cyclodesulfurization (Table 6 entry 5). The conditions for the two steps were optimized with broad substrate scopes, and the average conversion achieved 73%. Heinis' group synthesized the disulfide-cyclized peptide–DNA conjugates, which were utilized with bis-electrophile reagents for the construction of thioether-cyclized peptides (Table 6 entry 6).^75^ The NH_2_–(CH_2_)_6_–DNA underwent the condensation reaction with Fmoc–Cys(S–TMP)–OH, and the Fmoc group was deprotected. The Fmoc–glycine was introduced and subjected to deprotection. Finally, Fmoc–Cys(S–TMP)–OH was introduced, and the disulfide-cyclized peptide was formed at 10% piperidine and 5% DTT in water. The conditions were applied to other reactions, which the average conversion achieved 79% (Table 6 entry 7). Huang's group explored the synthesis of benzimidazoles on DNA *via* two-step reactions containing nitro reduction and cyclization (Table 6 entry 8).^76^ A new reduction condition of nitro was developed. The previous reductants RANEY® Ni with hydrazine utilized by chemists in Roche were replaced by Na_2_S_2_O_4_. Benzimidazoles were synthesized by amines and aldehydes under conventional conditions. The total average conversion achieved 84%. Du's group transformed the conventional synthesis of 1,2,4-oxadiazoles to the DNA-compatible reaction *via* a multistep reaction (Table 6 entry 9).^77^ DNA-conjugated aryl nitrile substrates reacted with hydroxylamine to form amidoxime. The ambient temperature was chosen rather than heating which avoiding the decomposition of DNA conjugates. The combination of buffer and coupling reagents for the *ortho*-acylation of amidoxime was screened, which indicated that pH 8.0 phosphate and PyAOP could promote the conversion near to 95%. DNA-conjugated amidoxime reacted with aromatic and aliphatic carboxylic acids to form the *O*-acylamidoxime with more than 90% average conversion. For the cyclodehydration of acylamidoximes, the combination of pH 9.5 borate (buffer) and *N*,*N*′-diisopropylethylamine (base) was chosen as the preferred reaction condition. A total of 54 examples for the synthesis of 1,2,4-oxadiazoles had an average conversion above 70%. Brunschweiger's group used the Pictet–Spengler reaction on DNA under strong acid catalysis (Table 6 entry 10).^64^ The group screened the reaction conditions and found that 1% trifluoroacetic acid could facilitate the conversion near to 100% in toluene, C_2_H_4_Cl_2_, MeCN, and CH_2_Cl_2_ for 18 h. However, DNA was degraded under the combined conditions of 10% trifluoroacetic acid and CH_2_Cl_2_ for 18 h. Recently, Lu and coworkers optimized the conditions of Pictet–Spengler reaction on DNA (Table 6 entry 11),^78^ which showed that the combination of i-PrOH/NMP (1 : 1) and pH 5.5 phosphate buffer was preferred. Two years later, Brunschweiger's group developed the synthesis of DNA-tagged heterocycles mediated by micellar Brønsted acid (Table 6 entry 12).^79^ The micelle-based acid catalyst was designed, and the sulfonic acid moieties were located in the internal hydrophobic pocket and the interface to the external hydrophilic shell. These acid nanoreactors promoted the DNA-conjugated aryl aldehydes to react with various substituted amines and olefins to form tetrahydroquinolines and aminoimidazopyridines *via* the Povarov and the Groebke–Blackburn–Bienaymé reactions. The development history of zirconium was shorter than that of palladium. Zirconium was utilized for condensation, Friedel–Crafts alkylation, intermolecular and intramolecular hydroamination, and asymmetric chiral catalysis reactions.^80–85^ Recently, scientists in GSK first developed the catalytic system of zirconium tetrakis(dodecyl sulfate) (Zr(DS)_4_) and ACN/H_2_O, which was utilized for the aminolysis of DNA-conjugated epoxides to form β-amino alcohols (Table 6 entry 13).^86^ Under the preferred conditions, most entries possessed 60–100% conversion, and all were applicable. A DEL containing 137 million compounds was also synthesized.

**Table 6:** Ring-closing and ring-opening reactions on DNA

Entry	DNA-compatible reactions	Conditions
1	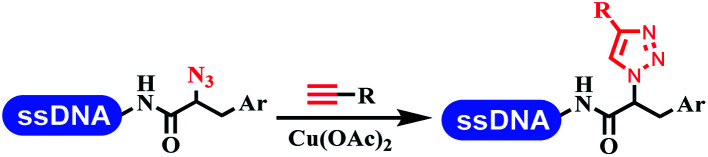	Na_2_CO_3_, sodium ascorbate, H_2_O, 35 °C, 3 h
2	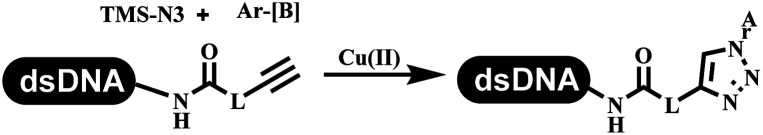	Sodium borate buffer, MDAc/H2O, 20 °C,16 h
3	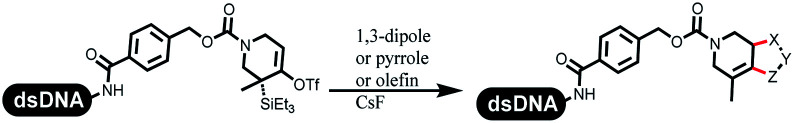	90% aq. DMSO, RT, 1 h
4	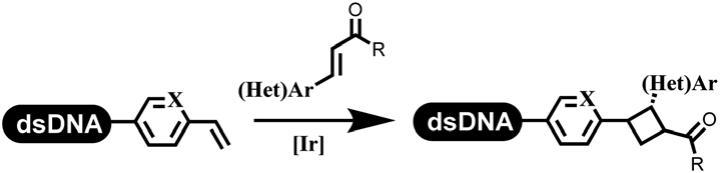	DMSO/H2O/glycerol (2 : 1 : 0.2), LED array
5	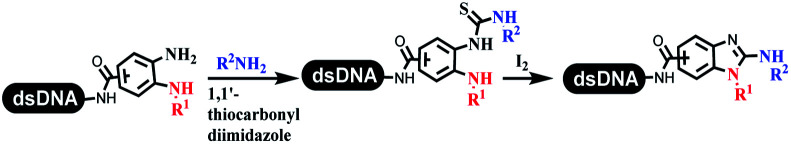	MeCN : H_2_O, DMSO, Et_3_N, borate buffer, RT, 16 h
6	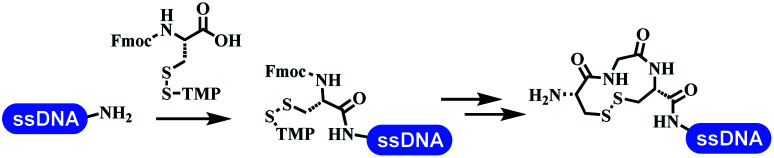	Borate buffer, H_2_O, RT
7	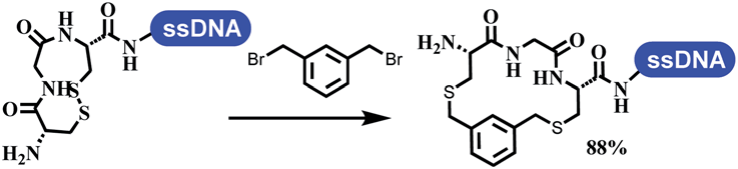	1.6 mM cyclization reagent
	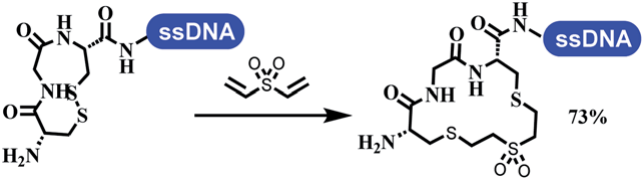	20% MeCN
	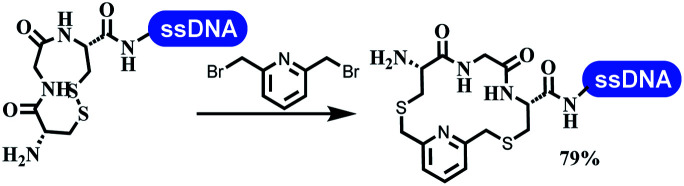	80% NH_4_HCO_3_ buffer, pH8
	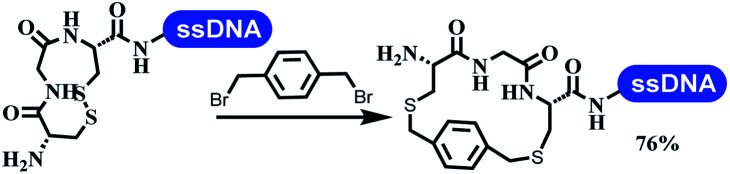	30 °C, 2 h; TCEP
	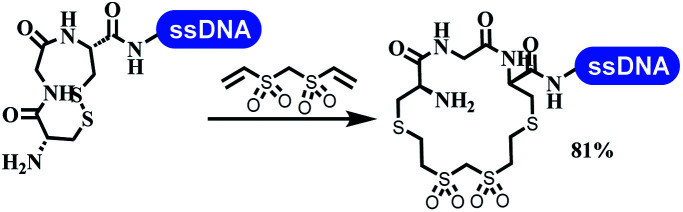	NH_4_HCO_3_ buffer, pH 8, RT, 1 h
8	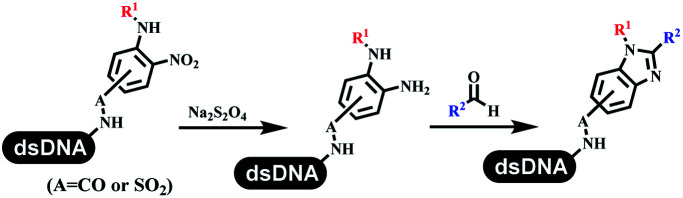	pH 9.5 borate buffer, viologen; 80 °C, 12 h
9	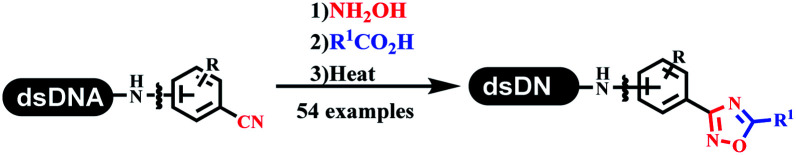	pH 8.2 borate, Na_2_CO_3_; pH 8.0 phose. Buffer, PyAOP; CH_3_CN, buffer, heat
10	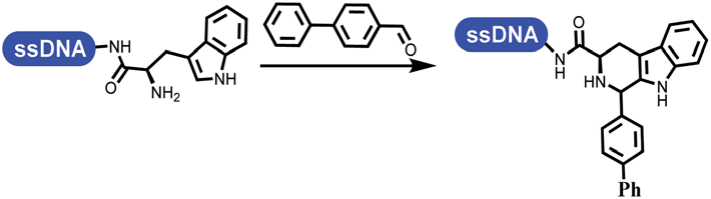	TFA, CH_2_Cl_2_,
11	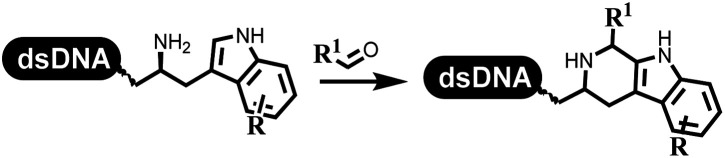	*i*-PrOH/NMP (1 : 1)
		pH 5.5 phosphate buffer
12	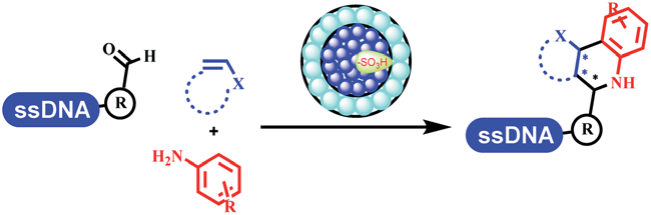	H_2_O, micellar catalysis
13	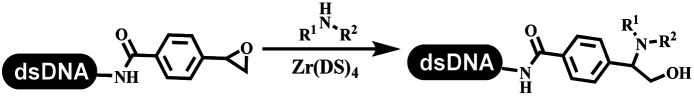	ACN/H_2_O, 50 °C

### Redox reactions

The chemists in the Roche Innovation Center used the RANEY® Ni with hydrazine to reduce the nitro to amine on DNA (Table 7 entry 1).^87^ Biocatalysis was implemented widely in drug development and production.^88^ Particularly, enzyme-catalyzed chemical reactions drew much attention due to high selectivity, mild reaction conditions (most in water), short reaction steps, effective atomic utilization, and renewability.^89^ GSK developed the first enzyme-catalyzed reactions on DNA, which were combined with traditional organic chemistry to synthesize the DNA-linked carbohydrate library.^90^ GSK used various galactose oxidases to oxidize hexoses C6–OH and form aldehydes on DNA, and the aldehydes transformed to other groups by hydrazone ligation or reductive amination (Table 7 entry 2). The metal-free reduction of nitro aromatics was catalyzed *via* the diboronic acid off DNA and reported by Wu and Zhou.^91,92^ Simmons's group used the efficient and facile reduction methodology for nitro on DNA (Table 7 entry 3).^93^ The preferred combination of Bases and solvents were sodium hydroxide and alcohol, which promoted the conversion up to 95%. DNA-linked aromatic and aliphatic nitro groups efficiently formed the desired amines, and the average conversion achieved above 82%. Finally, the method for DEL construction contained above 75 million compounds. Regularly, sulfonamides were synthesized through amines and sulfonyl chlorides in suitable organic solvents. However, transforming the routine method to the DNA-compatible reactions was difficult because sulfonyl chlorides were unstable in water. Peng's group used the DNA-linked amine to react with sodium benzenesulfinate or DNA-conjugated benzenesulfinic acid to react with amine to form sulfonamide under the oxidant catalysis, which avoided the limitation to sulfonyl chlorides (Table 7 entries 4 and 5).^94^ In the former reaction, the oxidant and the solvent were screened, and the combination of I_2_ and pH 9.5 buffer were preferred. The substrate scope was explored and indicated that DNA-conjugated aliphatic or (het)aryl amines and aryl sodium sulfinates could form the target sulfonamide molecules. The sulfonylation of aliphatic amines with phenyl sodium sulfinates showed the average conversion near to 86%. For the later reaction, the DNA-conjugated benzenesulfinic acid was used to react with diverse amines, and most conversion achieved above 80%. Neri's team optimized the reaction conditions to construct amide on DNA (Table 7 entry 6).^95^ Various coupling reagents were screened in eight amidation reactions, and results showed that the combination of 1-ethyl-3-(3-(dimethylamino)propyl)carbodiimide, 1-hydroxy-7-azabenzotriazole, and DIPEA was the preferred due to more than 90% average conversion. DNA-conjugated amines were used to react with diverse carboxylic acids, which provided average conversion greater than 75%, which 78% (423/543) of the carboxylic acid substrates were consumed.

**Table 7:** Redox and acylation reactions on DNA

Entry	DNA-compatible reactions	Conditions
1	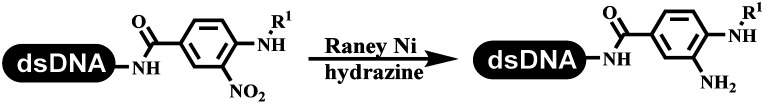	H_2_O, RT, 24 h, with shaking
2	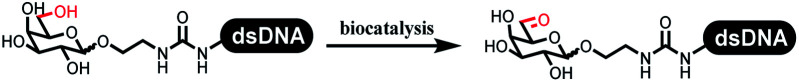	Enzyme
3	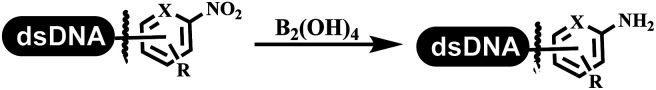	NaOH, aq. EtOH, 25 °C
4	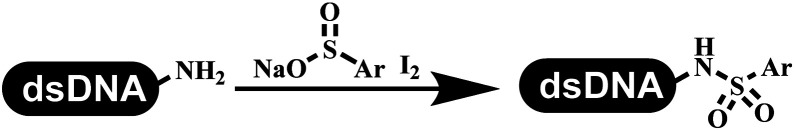	pH 9.5 buffer, RT, 16 h
5	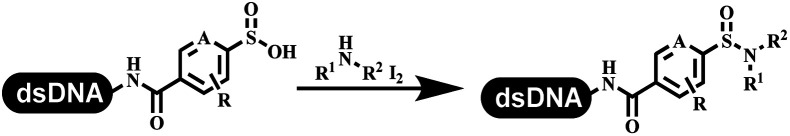	H_2_O, RT, 1 h
6	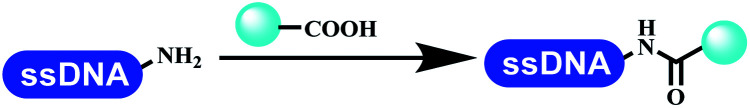	EDC/HOAt/DIPEA

### Hit/lead

Until June 2016, some excellent reviews summarized the use of DEL in identifying the hits to therapeutic targets.^5,6^ Here, we summarized some new ligands for targets, which were identified *via* the DEL from July 2016 to present (Table 8). Physicochemical properties, oral druggable space, and cell permeability of the hit/lead were predicted *via* the rules of Lipinski/Kihlberg.^96–98^ The possibility of studying these compounds against central nervous system (CNS) diseases was also predicted *via* the CNS Multiparameter Optimization (MPO) approach.^99,100^ Entry 1 compound could inhibit neurodegeneration *via* targeting SIRT6 (Sirtuin 6), but the MPO scored 3 (yellow) which means this compound need to be further optimized as a CNS drug. Entry 2 compound was the most potent inhibitor reported for PARP15 (poly(ADP-ribose) polymerase), and its' drug score was 0.45(yellow) which indicated the drug-conforming behavior need to be improved in further study, such a saturation was same as entry 3 and entry 4 compounds. Entry 5–8 compounds scored negatively in the items of drug score and drug likeness (yellow to red), and positively in other items (green). These results were contrary to entry 9 compound. Entry 10–12 compounds were perhaps difficult to use for CNS diseases treatment due to the low scores in items of drug score and MPO. To entry 13–15 compounds, Lipinski/Kihlberg, drug score and MPO three items were negative (yellow and red), these may attribute to higher molecular weight, more heteroatom and other uncertainties. Entry 16 compound possessed low scores in drug score and drug likeness (red), which were adverse to the drug-conforming behavior. However, for entry 5, 6, 10 and 11 compounds, Lipinski/Kihlberg, drug score, drug likeness and MPO four items were positive (green). Particularly for entry 5 compound, a BRD4 inhibitor, had a potent *vivo* activity (1.5 mg kg^−1^ in dog). To sum up, a compound was designed for CNS disease treatment, its four prediction items should be positive (green), if not, the CNS MPO should be negative. These should be seriously considered at the stage of molecules design in DEL, which could improve the efficiency drug discovery.

**Table 8:** Hits to therapeutic targets identified using DEL methods published between 2016 and 2020

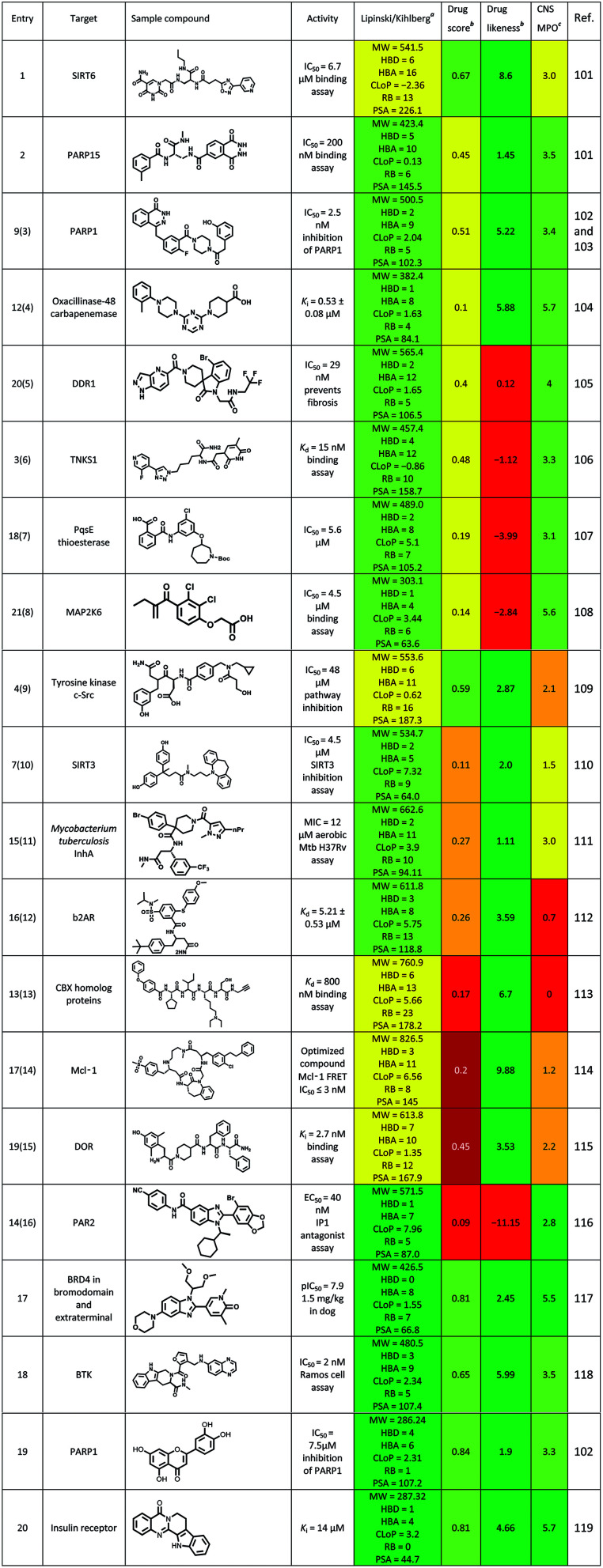

## Conclusion and outlook

Novel DNA-compatible reactions were constantly explored and developed. These reactions, which included transition and nontransition metal catalyses, photocatalysis, and biocatalysis, depend on catalysts. The DNA-conjugated reaction was not merely evaluated by the rate of conversion, but other important factors included the universality (passing rate) of synthon and the recovery rate of the DNA material. The DEL underwent initial exploration, technology accumulation, and preliminary application and achieved remarkable results in developing preclinical and clinical candidates. DEL was widely recognized as a method with great promise for lead generation and beyond due to high productivity and other benefits. However, challenges and limitations existed in DEL. These limitations included (1) limitations in types of BBs and reactions, (2) probable effect of oligonucleotide on binding affinity, (3) targeting of DNA-/RNA-binding proteins^4,120^ and (4) low drug ability of hit/lead (Table 8). The significant advantages of DEL included productivity, cost effectiveness, and efficiency, which cannot be surpassed by traditional HTS methods and other existing platform technologies. The limitations generated increased the chances to develop new encoding methods, such as DNA-compatible chemical reactions and other processes of DEL. For DEL, the future directions evidently focused on (1) expanding the chemical space and the diversity of DEL and (2) improving the druggability of hit/lead to further cut down the cost and time for drug discovery.

## Conflicts of interest

There are no conflicts to declare.

## Supplementary Material
